# 2-[1-(2-Hydr­oxy-3-methoxy­benz­yl)-1*H*-benzimidazol-2-yl]-6-methoxy­phenol methanol 1.13-solvate

**DOI:** 10.1107/S1600536809011192

**Published:** 2009-03-31

**Authors:** Mohammed H. Al-Douh, Hasnah Osman, Shafida A. Hamid, Reza Kia, Hoong-Kun Fun

**Affiliations:** a School of Chemical Sciences, Universiti Sains Malaysia, 11800 USM, Penang, Malaysia; b Kulliyyah of Science, International Islamic University Malaysia (IIUM), Jalan Istana, Bandar Indera Mahkota 25200 Kuantan, Pahang, Malaysia; c X-ray Crystallography Unit, School of Physics, Universiti Sains Malaysia, 11800 USM, Penang, Malaysia

## Abstract

In the main mol­ecule of the title compound, C_22_H_20_N_2_O_4_·1.13CH_4_O, the dihedral angles between the benzimidazole plane and the two benzene rings are 80.53 (10) and 82.76 (10)°. The solvent mol­ecules are disordered between three positions, with refined occupancies of 0.506 (13), 0.373 (13) and 0.249 (5). The crystal structure is stabilized by inter­molecular O—H⋯O, O—H⋯N and C—H⋯O hydrogen bonds. The crystal studied was a merohedral twin [BASF ratio of 0.917 (1)/0.083 (1)].

## Related literature

For related structures, see Al-Douh *et al.* (2006 ▶, 2009 ▶). For hydrogen-bond motifs, see Bernstein *et al.* (1995 ▶). For the stability of the temperature controller used for the data collection, see: Cosier & Glazer (1986 ▶).
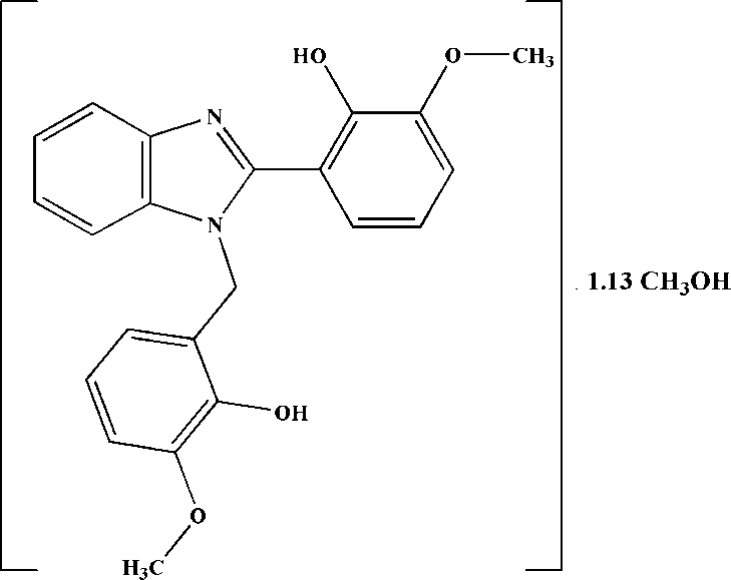


## Experimental

### 

#### Crystal data

C_22_H_20_N_2_O_4_·1.13CH_4_O
*M* *_r_* = 412.53
Monoclinic, 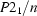

*a* = 7.2451 (1) Å
*b* = 11.1482 (2) Å
*c* = 26.2046 (5) Å
β = 90.010 (1)°
*V* = 2116.54 (6) Å^3^
*Z* = 4
Mo *K*α radiation
μ = 0.09 mm^−1^
*T* = 100 K
0.27 × 0.15 × 0.13 mm

#### Data collection

Bruker SMART APEXII CCD area-detector diffractometer
Absorption correction: multi-scan (**SADABS**; Bruker, 2005 ▶) *T*_min_ = 0.976, *T*_max_ = 0.988
18918 measured reflections
3940 independent reflections
3099 reflections with *I* > 2σ(*I*)
*R*_int_ = 0.040

#### Refinement

*R*[*F* ^2^ > 2σ(*F* ^2^)] = 0.050
*wR*(*F* ^2^) = 0.122
*S* = 1.09
3940 reflections
317 parameters
1 restraint
H-atom parameters constrained
Δρ_max_ = 0.28 e Å^−3^
Δρ_min_ = −0.23 e Å^−3^

### 

Data collection: *APEX2* (Bruker, 2005 ▶); cell refinement: *SAINT* (Bruker, 2005 ▶); data reduction: *SAINT*; program(s) used to solve structure: *SHELXTL* (Sheldrick, 2008 ▶); program(s) used to refine structure: *SHELXTL*; molecular graphics: *SHELXTL*; software used to prepare material for publication: *SHELXTL* and *PLATON* (Spek, 2009 ▶).

## Supplementary Material

Crystal structure: contains datablocks global, I. DOI: 10.1107/S1600536809011192/cv2535sup1.cif

Structure factors: contains datablocks I. DOI: 10.1107/S1600536809011192/cv2535Isup2.hkl

Additional supplementary materials:  crystallographic information; 3D view; checkCIF report

## Figures and Tables

**Table 1:** Hydrogen-bond geometry (Å, °)

*D*—H⋯*A*	*D*—H	H⋯*A*	*D*⋯*A*	*D*—H⋯*A*
O3—H3⋯O5*A*^i^	0.82	1.85	2.626 (5)	157
O5*A*—H5*A*1⋯N1^ii^	0.82	2.07	2.842 (5)	157
O1—H1⋯O3^iii^	0.82	2.19	2.956 (2)	155
C14—H14*B*⋯O2^iii^	0.97	2.46	3.335 (3)	150
C23*A*—H23*B*⋯O5*A*^iv^	0.96	2.57	3.215 (16)	125

Symmetry codes: (i) 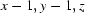; (ii) 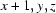; (iii) 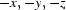; (iv) 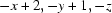.

**Fig. 1.**
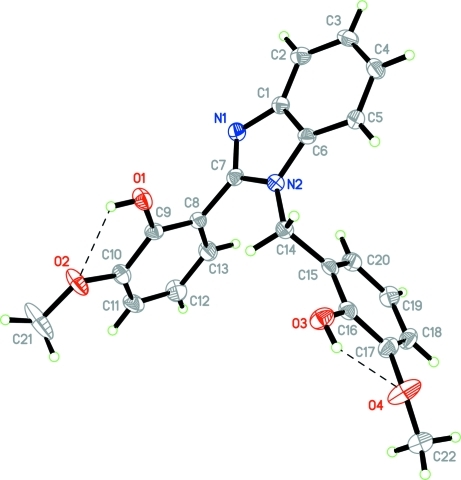
The molecular structure of the title compound, showing 50% probability displacement ellipsoids and the atomic numbering. The disordered methanol solvent molecules were omitted for clarity. Intramolecular hydrogen bonds are drawn as dashed lines.

## supplementary crystallographic information

### Comment

In continuation of our crystallographic study of benzimidazole derivatives (Al-Douh *et al.*, 2006, 2009), we present here the crystal structure of the title compound, (I).

In the title compound (Fig. 1), intramolecular O—H···O hydrogen bonds (Table 1) generate five-membered rings with *S(5)* ring motifs (Bernstein *et al.*, 1995). The dihedral angle between the two outer benzene rings is 75.18 (12)°. The benzimidazole plane and two outer benzene rings form dihedral angles 80.53 (10) and 82.76 (10)°, respectively. There are short intermolecular contacts C16···C21^vi^  of 3.209 (4) Å and C17···C21^vi^ of 3.308 (4) Å [symmetry code: (vi) 1 - *x*, -*y*, -*z*].

The crystal structure is stabilized by intermolecular O—H···O, O—H···N and C—H···O hydrogen bonds (Table 1).

### Experimental

A 100-mL, three-necked, round-bottomed flask is equipped with a nitrogen inlet adapter, rubber septum, glass stopper, and a magnetic stirring bar. The flask is charged with 5 mL of dichloromethane and (608.61 mg, 0.004 mol) of *o*-vanillin, and then is cooled in an ice-water bath while a solution of (216.29 mg, 0.002 mol) of *o*-phenylenediamine in 5 mL of dichloromethane is added dropwise via syringe over 15 min. After 30 min, 10 g of anhydrous magnesium sulfate is added in one portion. The ice-water bath is removed, and the reaction mixture is stirred at room temperature for 2hr. The resulting solution is allowed to cool to room temperature and then is cooled in an ice-water bath for 2 hr. Filtration provides the light yellowish powder. The single crystals suitable for *X*-ray diffraction were obtained by evaporation of methanol and dichloromethane (7:3) solvent at room temperature.

### Refinement

All H atoms were geometrically positioned (C—H 0.93–0.97 Å, O—H 0.82 Å) and refined in a riding model approximation, with *U*_iso_(H) = 1.2 or 1.5 (C, O). The methanol solvent molecules were treated as disordered over three positions with refined site-occupancies of 0.506 (13), 0.373 (13) and 0.249 (5) with SUMP command equal to 1.0 (1). The crystal studied was a twin with the refined BASF ratio of 0.917 (1)/0.083 (1). During the data collection, the temperature was controlled according to the literature procedure (Cosier & Glazer, 1986).

### Crystal data

**Table:**

C_22_H_20_N_2_O_4_·1.13CH_4_O	*F*(000) = 873
*M**_r_* = 412.53	*D*_x_ = 1.295 Mg m^−^^3^
Monoclinic, *P*2_1_/*n*	Mo *K*α radiation, λ = 0.71073 Å
Hall symbol: -P 2yn	Cell parameters from 4846 reflections
*a* = 7.2451 (1) Å	θ = 2.9–26.4°
*b* = 11.1482 (2) Å	µ = 0.09 mm^−^^1^
*c* = 26.2046 (5) Å	*T* = 100 K
β = 90.010 (1)°	Block, colourless
*V* = 2116.54 (6) Å^3^	0.27 × 0.15 × 0.13 mm
*Z* = 4	

### Data collection

**Table:**

Bruker SMART APEXII CCD area-detector diffractometer	3940 independent reflections
Radiation source: fine-focus sealed tube	3099 reflections with *I* > 2σ(*I*)
graphite	*R*_int_ = 0.040
φ and ω scans	θ_max_ = 25.5°, θ_min_ = 1.8°
Absorption correction: multi-scan (*SADABS*; Bruker, 2005)	*h* = −8→8
*T*_min_ = 0.976, *T*_max_ = 0.988	*k* = −12→13
18918 measured reflections	*l* = −31→28

### Refinement

**Table:**

Refinement on *F*^2^	Primary atom site location: structure-invariant direct methods
Least-squares matrix: full	Secondary atom site location: difference Fourier map
*R*[*F*^2^ > 2σ(*F*^2^)] = 0.050	Hydrogen site location: inferred from neighbouring sites
*wR*(*F*^2^) = 0.122	H-atom parameters constrained
*S* = 1.09	*w* = 1/[σ^2^(*F*_o_^2^) + (0.0415*P*)^2^ + 1.5448*P*] where *P* = (*F*_o_^2^ + 2*F*_c_^2^)/3
3940 reflections	(Δ/σ)_max_ < 0.001
317 parameters	Δρ_max_ = 0.28 e Å^−^^3^
1 restraint	Δρ_min_ = −0.23 e Å^−^^3^

### Special details

**Table:**

Experimental. The crystal was placed in the cold stream of an Oxford Cyrosystems Cobra open-flow nitrogen cryostat (Cosier & Glazer, 1986) operating at 100.0 (1) K.
Geometry. All e.s.d.'s (except the e.s.d. in the dihedral angle between two l.s. planes) are estimated using the full covariance matrix. The cell e.s.d.'s are taken into account individually in the estimation of e.s.d.'s in distances, angles and torsion angles; correlations between e.s.d.'s in cell parameters are only used when they are defined by crystal symmetry. An approximate (isotropic) treatment of cell e.s.d.'s is used for estimating e.s.d.'s involving l.s. planes.
Refinement. Refinement of *F*^2^ against ALL reflections. The weighted *R*-factor *wR* and goodness of fit *S* are based on *F*^2^, conventional *R*-factors *R* are based on *F*, with *F* set to zero for negative *F*^2^. The threshold expression of *F*^2^ > σ(*F*^2^) is used only for calculating *R*-factors(gt) *etc*. and is not relevant to the choice of reflections for refinement. *R*-factors based on *F*^2^ are statistically about twice as large as those based on *F*, and *R*- factors based on ALL data will be even larger.

### Fractional atomic coordinates and isotropic or equivalent isotropic displacement parameters (Å^2^)

**Table:**

	*x*	*y*	*z*	*U*_iso_*/*U*_eq_	Occ. (<1)
O1	0.0363 (2)	0.18739 (17)	0.00815 (6)	0.0272 (4)	
H1	0.0385	0.1891	−0.0231	0.041*	
O2	0.3146 (2)	0.15819 (18)	−0.05634 (6)	0.0346 (5)	
O3	0.0521 (3)	−0.24674 (15)	0.09905 (6)	0.0282 (4)	
H3	0.0664	−0.3196	0.0971	0.042*	
O4	0.2424 (3)	−0.39894 (16)	0.15953 (8)	0.0437 (6)	
N1	0.0259 (3)	0.31698 (18)	0.13518 (7)	0.0220 (5)	
N2	−0.0044 (3)	0.11719 (17)	0.13178 (7)	0.0196 (5)	
C1	−0.1122 (3)	0.2812 (2)	0.16904 (9)	0.0199 (5)	
C2	−0.2243 (3)	0.3492 (2)	0.20124 (9)	0.0237 (6)	
H2A	−0.2161	0.4324	0.2019	0.028*	
C3	−0.3483 (3)	0.2886 (2)	0.23220 (9)	0.0253 (6)	
H3A	−0.4232	0.3321	0.2543	0.030*	
C4	−0.3634 (4)	0.1640 (2)	0.23092 (9)	0.0255 (6)	
H4A	−0.4479	0.1264	0.2523	0.031*	
C5	−0.2560 (3)	0.0947 (2)	0.19875 (9)	0.0229 (6)	
H5A	−0.2666	0.0116	0.1977	0.027*	
C6	−0.1313 (3)	0.1562 (2)	0.16799 (8)	0.0195 (5)	
C7	0.0860 (3)	0.2171 (2)	0.11421 (9)	0.0198 (5)	
C8	0.2427 (3)	0.2062 (2)	0.07811 (9)	0.0196 (5)	
C9	0.2130 (3)	0.1894 (2)	0.02649 (9)	0.0202 (5)	
C10	0.3634 (4)	0.1738 (2)	−0.00633 (9)	0.0233 (6)	
C11	0.5417 (4)	0.1767 (2)	0.01214 (10)	0.0289 (6)	
H11A	0.6411	0.1665	−0.0099	0.035*	
C12	0.5715 (4)	0.1951 (3)	0.06398 (10)	0.0324 (7)	
H12A	0.6913	0.1981	0.0766	0.039*	
C13	0.4233 (3)	0.2090 (2)	0.09689 (10)	0.0281 (6)	
H13A	0.4441	0.2203	0.1316	0.034*	
C14	0.0209 (3)	−0.0061 (2)	0.11471 (9)	0.0203 (5)	
H14A	0.0840	−0.0054	0.0821	0.024*	
H14B	−0.0994	−0.0423	0.1095	0.024*	
C15	0.1298 (3)	−0.0828 (2)	0.15162 (9)	0.0193 (5)	
C16	0.1386 (3)	−0.2048 (2)	0.14165 (9)	0.0210 (5)	
C17	0.2424 (4)	−0.2802 (2)	0.17332 (10)	0.0256 (6)	
C18	0.3361 (4)	−0.2327 (2)	0.21462 (10)	0.0267 (6)	
H18A	0.4048	−0.2825	0.2358	0.032*	
C19	0.3274 (3)	−0.1111 (2)	0.22427 (10)	0.0263 (6)	
H19A	0.3906	−0.0793	0.2520	0.032*	
C20	0.2259 (3)	−0.0364 (2)	0.19313 (9)	0.0241 (6)	
H20A	0.2217	0.0454	0.1999	0.029*	
C21	0.4624 (5)	0.1568 (4)	−0.09260 (11)	0.0588 (11)	
H21A	0.4126	0.1590	−0.1265	0.088*	
H21B	0.5337	0.0849	−0.0883	0.088*	
H21C	0.5400	0.2255	−0.0873	0.088*	
C22	0.4019 (4)	−0.4674 (3)	0.17089 (12)	0.0399 (7)	
H22A	0.5094	−0.4261	0.1587	0.060*	
H22B	0.3931	−0.5443	0.1546	0.060*	
H22C	0.4112	−0.4783	0.2071	0.060*	
O5A	1.0245 (11)	0.5263 (4)	0.0733 (2)	0.034 (2)	0.506 (13)
H5A1	1.0285	0.4805	0.0978	0.051*	0.506 (13)
C23A	0.8593 (19)	0.5039 (7)	0.0444 (6)	0.082 (5)	0.506 (13)
H23A	0.8527	0.4203	0.0358	0.123*	0.506 (13)
H23B	0.8616	0.5507	0.0136	0.123*	0.506 (13)
H23C	0.7534	0.5257	0.0643	0.123*	0.506 (13)
O5B	0.9164 (17)	0.5416 (6)	0.0975 (4)	0.048 (4)	0.373 (13)
H5B	0.9997	0.4977	0.0874	0.072*	0.373 (13)
C23B	0.739 (3)	0.5162 (11)	0.0664 (4)	0.064 (4)	0.373 (13)
H23D	0.7633	0.4547	0.0417	0.096*	0.373 (13)
H23E	0.7007	0.5881	0.0493	0.096*	0.373 (13)
H23F	0.6429	0.4900	0.0891	0.096*	0.373 (13)
O5C	0.718 (2)	0.4931 (11)	0.0088 (4)	0.082 (4)	0.249 (5)
H5C	0.7138	0.4829	−0.0222	0.123*	0.249 (5)
C23C	0.537 (3)	0.5098 (15)	0.0278 (8)	0.092 (7)	0.249 (5)
H23G	0.4542	0.4569	0.0102	0.139*	0.249 (5)
H23H	0.5401	0.4894	0.0633	0.139*	0.249 (5)
H23I	0.4957	0.5911	0.0239	0.139*	0.249 (5)

### Atomic displacement parameters (Å^2^)

**Table:**

	*U*^11^	*U*^22^	*U*^33^	*U*^12^	*U*^13^	*U*^23^
O1	0.0169 (9)	0.0456 (11)	0.0192 (9)	−0.0011 (8)	0.0000 (7)	−0.0004 (8)
O2	0.0215 (10)	0.0621 (13)	0.0202 (9)	−0.0080 (9)	0.0070 (8)	−0.0083 (9)
O3	0.0329 (10)	0.0216 (9)	0.0302 (10)	0.0024 (8)	−0.0068 (8)	−0.0033 (7)
O4	0.0460 (13)	0.0217 (10)	0.0633 (14)	0.0073 (9)	−0.0233 (11)	−0.0015 (9)
N1	0.0227 (11)	0.0222 (11)	0.0212 (10)	−0.0008 (9)	0.0010 (9)	0.0005 (9)
N2	0.0197 (11)	0.0203 (11)	0.0188 (10)	0.0011 (9)	0.0016 (9)	0.0001 (8)
C1	0.0194 (12)	0.0224 (13)	0.0179 (11)	0.0008 (10)	−0.0033 (10)	−0.0011 (10)
C2	0.0222 (13)	0.0258 (14)	0.0231 (13)	0.0033 (11)	−0.0044 (11)	−0.0039 (11)
C3	0.0197 (13)	0.0343 (15)	0.0220 (12)	0.0064 (12)	0.0010 (11)	−0.0048 (11)
C4	0.0207 (13)	0.0348 (15)	0.0210 (12)	−0.0006 (12)	0.0027 (11)	0.0019 (11)
C5	0.0234 (13)	0.0231 (13)	0.0220 (12)	0.0017 (11)	0.0002 (11)	0.0036 (10)
C6	0.0175 (12)	0.0248 (13)	0.0163 (11)	0.0047 (11)	−0.0010 (10)	−0.0020 (10)
C7	0.0201 (13)	0.0212 (13)	0.0182 (11)	0.0015 (11)	−0.0030 (10)	0.0011 (10)
C8	0.0196 (12)	0.0187 (12)	0.0204 (12)	0.0006 (10)	0.0020 (10)	0.0009 (10)
C9	0.0157 (12)	0.0203 (13)	0.0246 (12)	0.0003 (10)	0.0002 (10)	0.0002 (10)
C10	0.0233 (13)	0.0258 (14)	0.0208 (12)	−0.0035 (11)	0.0013 (11)	−0.0023 (10)
C11	0.0192 (13)	0.0366 (16)	0.0310 (14)	0.0028 (12)	0.0064 (11)	−0.0009 (12)
C12	0.0162 (13)	0.0496 (18)	0.0314 (15)	0.0010 (13)	−0.0038 (12)	0.0039 (13)
C13	0.0231 (14)	0.0394 (16)	0.0219 (13)	0.0032 (12)	−0.0023 (11)	0.0013 (12)
C14	0.0204 (12)	0.0216 (13)	0.0188 (12)	0.0004 (11)	0.0014 (10)	−0.0003 (10)
C15	0.0154 (12)	0.0232 (13)	0.0194 (12)	−0.0001 (10)	0.0051 (10)	0.0002 (10)
C16	0.0170 (12)	0.0248 (13)	0.0212 (12)	−0.0005 (11)	0.0032 (10)	0.0004 (10)
C17	0.0237 (13)	0.0208 (13)	0.0324 (14)	−0.0007 (11)	−0.0003 (12)	0.0039 (11)
C18	0.0228 (14)	0.0280 (15)	0.0292 (14)	0.0028 (12)	−0.0023 (12)	0.0102 (11)
C19	0.0222 (14)	0.0315 (15)	0.0253 (13)	−0.0009 (12)	−0.0042 (11)	0.0013 (11)
C20	0.0215 (13)	0.0253 (14)	0.0254 (13)	−0.0003 (11)	0.0016 (11)	−0.0013 (11)
C21	0.0388 (19)	0.110 (3)	0.0275 (16)	−0.026 (2)	0.0169 (15)	−0.0232 (18)
C22	0.0440 (18)	0.0312 (16)	0.0445 (17)	0.0119 (14)	0.0048 (15)	0.0037 (13)
O5A	0.056 (4)	0.016 (2)	0.030 (3)	−0.007 (2)	−0.017 (3)	0.0041 (17)
C23A	0.108 (9)	0.024 (4)	0.114 (10)	−0.011 (5)	−0.075 (8)	0.012 (5)
O5B	0.064 (7)	0.024 (4)	0.056 (6)	−0.014 (4)	−0.034 (6)	0.011 (3)
C23B	0.109 (12)	0.044 (6)	0.039 (6)	−0.029 (7)	0.002 (7)	−0.010 (5)
O5C	0.125 (12)	0.067 (8)	0.053 (7)	−0.011 (8)	−0.017 (8)	0.022 (6)
C23C	0.090 (15)	0.050 (10)	0.137 (18)	−0.013 (10)	−0.052 (14)	0.043 (11)

### Geometric parameters (Å, °)

**Table:**

O1—C9	1.367 (3)	C14—H14A	0.9700
O1—H1	0.8200	C14—H14B	0.9700
O2—C10	1.368 (3)	C15—C16	1.387 (3)
O2—C21	1.432 (3)	C15—C20	1.391 (3)
O3—C16	1.363 (3)	C16—C17	1.400 (4)
O3—H3	0.8200	C17—C18	1.383 (4)
O4—C17	1.372 (3)	C18—C19	1.380 (4)
O4—C22	1.416 (3)	C18—H18A	0.9300
N1—C7	1.316 (3)	C19—C20	1.379 (4)
N1—C1	1.396 (3)	C19—H19A	0.9300
N2—C7	1.371 (3)	C20—H20A	0.9300
N2—C6	1.391 (3)	C21—H21A	0.9600
N2—C14	1.457 (3)	C21—H21B	0.9600
C1—C2	1.395 (3)	C21—H21C	0.9600
C1—C6	1.401 (3)	C22—H22A	0.9600
C2—C3	1.386 (4)	C22—H22B	0.9600
C2—H2A	0.9300	C22—H22C	0.9600
C3—C4	1.394 (4)	O5A—C23A	1.439 (9)
C3—H3A	0.9300	O5A—H5A1	0.8200
C4—C5	1.383 (4)	C23A—H23A	0.9600
C4—H4A	0.9300	C23A—H23B	0.9600
C5—C6	1.392 (3)	C23A—H23C	0.9600
C5—H5A	0.9300	O5B—C23B	1.547 (16)
C7—C8	1.483 (3)	O5B—H5B	0.8200
C8—C9	1.382 (3)	C23B—H23D	0.9600
C8—C13	1.398 (4)	C23B—H23E	0.9600
C9—C10	1.400 (3)	C23B—H23F	0.9600
C10—C11	1.380 (4)	O5C—C23C	1.41 (2)
C11—C12	1.390 (4)	O5C—H5C	0.8200
C11—H11A	0.9300	C23C—C23C^i^	1.57 (4)
C12—C13	1.386 (4)	C23C—H23G	0.9600
C12—H12A	0.9300	C23C—H23H	0.9597
C13—H13A	0.9300	C23C—H23I	0.9602
C14—C15	1.513 (3)		
			
C9—O1—H1	109.5	C16—C15—C14	117.3 (2)
C10—O2—C21	116.3 (2)	C20—C15—C14	123.4 (2)
C16—O3—H3	109.5	O3—C16—C15	118.0 (2)
C17—O4—C22	117.7 (2)	O3—C16—C17	121.8 (2)
C7—N1—C1	105.1 (2)	C15—C16—C17	120.2 (2)
C7—N2—C6	106.90 (19)	O4—C17—C18	125.2 (2)
C7—N2—C14	127.1 (2)	O4—C17—C16	115.0 (2)
C6—N2—C14	126.0 (2)	C18—C17—C16	119.8 (2)
C2—C1—N1	130.3 (2)	C19—C18—C17	119.8 (2)
C2—C1—C6	119.7 (2)	C19—C18—H18A	120.1
N1—C1—C6	110.0 (2)	C17—C18—H18A	120.1
C3—C2—C1	117.8 (2)	C20—C19—C18	120.6 (2)
C3—C2—H2A	121.1	C20—C19—H19A	119.7
C1—C2—H2A	121.1	C18—C19—H19A	119.7
C2—C3—C4	121.5 (2)	C19—C20—C15	120.4 (2)
C2—C3—H3A	119.3	C19—C20—H20A	119.8
C4—C3—H3A	119.3	C15—C20—H20A	119.8
C5—C4—C3	121.8 (2)	O2—C21—H21A	109.5
C5—C4—H4A	119.1	O2—C21—H21B	109.5
C3—C4—H4A	119.1	H21A—C21—H21B	109.5
C4—C5—C6	116.3 (2)	O2—C21—H21C	109.5
C4—C5—H5A	121.8	H21A—C21—H21C	109.5
C6—C5—H5A	121.8	H21B—C21—H21C	109.5
N2—C6—C5	132.1 (2)	O4—C22—H22A	109.5
N2—C6—C1	105.0 (2)	O4—C22—H22B	109.5
C5—C6—C1	122.9 (2)	H22A—C22—H22B	109.5
N1—C7—N2	112.9 (2)	O4—C22—H22C	109.5
N1—C7—C8	126.1 (2)	H22A—C22—H22C	109.5
N2—C7—C8	120.9 (2)	H22B—C22—H22C	109.5
C9—C8—C13	119.5 (2)	O5A—C23A—H23A	109.5
C9—C8—C7	121.1 (2)	O5A—C23A—H23B	109.5
C13—C8—C7	119.4 (2)	H23A—C23A—H23B	109.5
O1—C9—C8	119.5 (2)	O5A—C23A—H23C	109.5
O1—C9—C10	120.7 (2)	H23A—C23A—H23C	109.5
C8—C9—C10	119.8 (2)	H23B—C23A—H23C	109.5
O2—C10—C11	125.5 (2)	C23B—O5B—H5B	109.5
O2—C10—C9	113.8 (2)	O5B—C23B—H23D	109.5
C11—C10—C9	120.7 (2)	O5B—C23B—H23E	109.5
C10—C11—C12	119.4 (2)	H23D—C23B—H23E	109.5
C10—C11—H11A	120.3	O5B—C23B—H23F	109.5
C12—C11—H11A	120.3	H23D—C23B—H23F	109.5
C13—C12—C11	120.3 (2)	H23E—C23B—H23F	109.5
C13—C12—H12A	119.8	O5B—C23B—H23H	151.5
C11—C12—H12A	119.8	H23D—C23B—H23H	89.8
C12—C13—C8	120.2 (2)	H23E—C23B—H23H	82.0
C12—C13—H13A	119.9	O5C—C23C—C23C^i^	88 (2)
C8—C13—H13A	119.9	O5C—C23C—H23G	109.3
N2—C14—C15	113.7 (2)	O5C—C23C—H23H	106.9
N2—C14—H14A	108.8	C23C^i^—C23C—H23H	151.2
C15—C14—H14A	108.8	H23G—C23C—H23H	109.5
N2—C14—H14B	108.8	O5C—C23C—H23I	112.2
C15—C14—H14B	108.8	C23C^i^—C23C—H23I	85.8
H14A—C14—H14B	107.7	H23G—C23C—H23I	109.5
C16—C15—C20	119.2 (2)	H23H—C23C—H23I	109.5
			
C7—N1—C1—C2	−179.5 (2)	C21—O2—C10—C9	172.4 (3)
C7—N1—C1—C6	0.7 (3)	O1—C9—C10—O2	0.6 (3)
N1—C1—C2—C3	−178.3 (2)	C8—C9—C10—O2	−180.0 (2)
C6—C1—C2—C3	1.6 (3)	O1—C9—C10—C11	179.5 (2)
C1—C2—C3—C4	−0.9 (4)	C8—C9—C10—C11	−1.0 (4)
C2—C3—C4—C5	−0.1 (4)	O2—C10—C11—C12	179.0 (3)
C3—C4—C5—C6	0.4 (4)	C9—C10—C11—C12	0.2 (4)
C7—N2—C6—C5	−178.4 (3)	C10—C11—C12—C13	0.7 (4)
C14—N2—C6—C5	3.9 (4)	C11—C12—C13—C8	−0.8 (4)
C7—N2—C6—C1	1.5 (2)	C9—C8—C13—C12	0.0 (4)
C14—N2—C6—C1	−176.2 (2)	C7—C8—C13—C12	177.9 (2)
C4—C5—C6—N2	−179.8 (2)	C7—N2—C14—C15	104.5 (3)
C4—C5—C6—C1	0.3 (4)	C6—N2—C14—C15	−78.2 (3)
C2—C1—C6—N2	178.7 (2)	N2—C14—C15—C16	171.5 (2)
N1—C1—C6—N2	−1.4 (3)	N2—C14—C15—C20	−11.3 (3)
C2—C1—C6—C5	−1.4 (4)	C20—C15—C16—O3	−176.2 (2)
N1—C1—C6—C5	178.5 (2)	C14—C15—C16—O3	1.2 (3)
C1—N1—C7—N2	0.3 (3)	C20—C15—C16—C17	0.4 (3)
C1—N1—C7—C8	−175.3 (2)	C14—C15—C16—C17	177.8 (2)
C6—N2—C7—N1	−1.2 (3)	C22—O4—C17—C18	−30.2 (4)
C14—N2—C7—N1	176.5 (2)	C22—O4—C17—C16	149.2 (2)
C6—N2—C7—C8	174.7 (2)	O3—C16—C17—O4	−3.0 (3)
C14—N2—C7—C8	−7.6 (4)	C15—C16—C17—O4	−179.5 (2)
N1—C7—C8—C9	−104.0 (3)	O3—C16—C17—C18	176.5 (2)
N2—C7—C8—C9	80.6 (3)	C15—C16—C17—C18	0.0 (4)
N1—C7—C8—C13	78.1 (3)	O4—C17—C18—C19	179.2 (2)
N2—C7—C8—C13	−97.3 (3)	C16—C17—C18—C19	−0.3 (4)
C13—C8—C9—O1	−179.6 (2)	C17—C18—C19—C20	0.1 (4)
C7—C8—C9—O1	2.5 (4)	C18—C19—C20—C15	0.4 (4)
C13—C8—C9—C10	0.9 (4)	C16—C15—C20—C19	−0.6 (3)
C7—C8—C9—C10	−177.0 (2)	C14—C15—C20—C19	−177.8 (2)
C21—O2—C10—C11	−6.5 (4)		

Symmetry codes: (i) −*x*+1, −*y*+1, −*z*.

### Hydrogen-bond geometry (Å, °)

**Table:**

*D*—H···*A*	*D*—H	H···*A*	*D*···*A*	*D*—H···*A*
O1—H1···O2	0.82	2.21	2.651 (2)	114
O3—H3···O4	0.82	2.25	2.700 (3)	114
O3—H3···O5A^ii^	0.82	1.85	2.626 (5)	157
O5A—H5A1···N1^iii^	0.82	2.07	2.842 (5)	157
O1—H1···O3^iv^	0.82	2.19	2.956 (2)	155
C14—H14B···O2^iv^	0.97	2.46	3.335 (3)	150
C23A—H23B···O5A^v^	0.96	2.57	3.215 (16)	125

Symmetry codes: (ii) *x*−1, *y*−1, *z*; (iii) *x*+1, *y*, *z*; (iv) −*x*, −*y*, −*z*; (v) −*x*+2, −*y*+1, −*z*.
